# Comparison of Antibodies with Amylase Activity from Cerebrospinal Fluid and Serum of Patients with Multiple Sclerosis

**DOI:** 10.1371/journal.pone.0154688

**Published:** 2016-05-19

**Authors:** Vasilii B. Doronin, Taisiya A. Parkhomenko, Massimiliano Castellazzi, Edward Cesnik, Valentina N. Buneva, Enrico Granieri, Georgy A. Nevinsky

**Affiliations:** 1 Novosibirsk Medical University, Ministry of Public Health of Russian Federation, Novosibirsk, Russia; 2 Institute of Chemical Biology and Fundamental Medicine, Russian Academy of Sciences, Siberian Division, Novosibirsk, Russia; 3 Multiple Sclerosis Center, Department of Neurology, Ferrara University, Ferrara, Italy; 4 Novosibirsk State University, Novosibirsk, Russia; University of Missouri-Kansas City, UNITED STATES

## Abstract

We have recently shown that IgGs from serum and cerebrospinal fluid (CSF) of MS patients are active in hydrolysis of DNA and myelin basic protein. According to literature data, anti-DNA and anti-MBP abzymes may promote important neuropathologic mechanisms in this chronic inflammatory disorder and in MS pathogenesis development. At the same time, the involvement of antibodies with amylase activity in the pathogenesis of any autoimmune disease has not yet been identified. Electrophoretically and immunologically homogeneous IgGs were obtained by a sequential affinity chromatography of the CSF proteins on protein G-Sepharose and FPLC gel filtration. We are able to present the first unpredictable evidence showing that IgGs from CSF possess amylase activity and efficiently hydrolyze maltoheptaose; their average specific Ab activity is ~30-fold higher than that of antibodies from sera of the same MS patients. Specific average RA (SAA) for IgGs from healthy volunteers was approximately ~1000 lower than that for MS patients. In addition, it was shown that a relative SAA of total proteins of CSF (including Abs) ~15-fold lower than that for purified IgGs, while the relative SAA of the total sera protein is higher than that of sera IgGs by a factor of 1033. This result speaks in favor of the fact that amylolytic activity of CSF proteins is mainly caused by the activity of amylase abzymes. One cannot exclude, that amylase abzymes of CSF can play a, as yet unknown, role in the pathogenesis of MS. Some possible reasons of these findings are discussed.

## Introduction

Multiple sclerosis (MS) is a chronic demyelinating pathology of the central nervous system presenting a serious medical and social problem. Its etiology remains unclear, and the most valid theory of its pathogenesis assigns the main role in the destruction of the myelin-proteolipid shell of axons to inflammation, related to autoimmune reactions ([1], and refs therein). Although the T-cell immune system plays a leading role in MS pathogenesis, the normal functioning of the B-cell system is also important for the development of the disease. An enhanced synthesis of immunoglobulins (usually IgGs), their free light chains and of a polyspecific DNA binding Abs interacting with phospholipids can be observed in MS patients [1].

SLE is a systemic autoimmune polyetiologically spread disease characterized by the disorganization of conjunctive tissues with a paramount damage to skin and visceral capillaries [2]. The polyetiologic and polysyndromic character of SLE leads to highly variable manifestations of this disease in terms of many biochemical, immunological and clinical indices. SLE is usually considered to be related to the patient’s autoimmunization with DNA, since sera of such patients usually contain DNA and anti-DNA Abs in high concentrations [3]. It should be mentioned that SLE and MS demonstrated some similarity in the development of the same medical, biochemical and immunological indexes.

Artificial abzymes (catalytic Abs against transition state analogues of chemical reactions) and natural abzymes are novel biological catalysts that have attracted a lot of interest in recent years (reviewed in [4–8]). Artificial abzymes are abzymes against analogues of transition states of catalytic reactions [4–8] or antiidiotypic Abs induced by a primary antigen, which may show some of their features including the catalytic activity (for review also see [9–14]). During the past two decades it has become clear that auto-antibodies (auto-Abs) from sera of patients with different autoimmune diseases can possess enzymatic activities and that their occurrence is a distinctive feature of autoimmune diseases (reviewed in [9–14]). Different abzymes may play a significant role in forming specific pathogenic patterns and clinical settings in different autoimmune conditions through their broadened auto-Ab properties. Patients with autoimmune diseases produce Abs to nucleoprotein complexes, DNA and enzymes that participate in nucleic acid metabolism [9–14].

Natural abzymes hydrolyzing DNA, RNA, oligopeptides and proteins are present in serum of patients with several autoimmune and viral diseases (reviewed in [14–17]). Healthy humans do not develop abzymes with detectable DNase and RNase activities, their levels being usually on the borderline of sensitivity of the detection methods [14–17].

It has recently been shown that myelin basic protein (MBP)-hydrolyzing [17–25] and DNase [26–29] activities are intrinsic properties of IgGs, IgMs, and IgAs from sera of MS and SLE patients, autoimmune SLE and experimental autoimmune encephalomyelitis (EAE) mice [30, 31]. Recognition and degradation of MBP peptides by serum auto-Abs were confirmed as a novel biomarker for MS [22, 23]. The established MS drug Copaxone appears to be a specific inhibitor of MBP-hydrolyzing abzyme activity [22, 23].

For a long time it was not clear whether DNA- and MBP-hydrolyzing antibodies can exist only in the blood of patients with MS and SLE, or if they may also be found in the cerebrospinal fluid. We have recently shown, that the average content of IgGs in their sera is about 195-fold higher than that in their CSF [32, 33]. We presented first evidence showing that IgGs from CSF efficiently hydrolyze MBP and DNA and that their average specific catalytic activity is unpredictably ~49 and 54-fold, respectively higher than that of Abs from sera of the same MS patients [32, 33].

IgGs and IgMs from sera of patients with several autoimmune diseases [34–38] and sIgAs from human breast milk [39] possess amylase activity, however the maximal activity was observed for Abs from sera of patients with MS [34, 37, 38] and SLE [34, 36]. The ability of presence of MBP- and DNA-hydrolyzing antibodies in the cerebrospinal fluid of MS patients may be related to the fact that anti-MBP and anti-DNA Abs may play an important role in the pathogenesis of this disease. In MS, the protease activity of anti-MBP abzymes can attack MBP of the myelin-proteolipid shell of axons [37, 38]. Similarly to SLE, high-affinity anti-DNA Abs has recently been identified as the major component of intrathecal IgGs in MS patients’ brains and in the cells of their cerebrospinal fluid [40]. In addition, DNase abzymes of MS patients [17, 18] similarly to SLE patients [41] are cytotoxic and induce apoptosis, which can play an important role in SLE and MS pathogenesis. At the same time, the involvement of antibodies with amylase activity in the pathogenesis of any autoimmune diseases has not yet been identified. However, a possible role of amylase abzymes in the development of autoimmune reactions could not be excluded. According to our point of view, in the pathogenesis of several autoimmune diseases several combinations of abzymes with various different catalytic activities can play an important role. Taking this into account, the analysis of amylase abzymes in the cerebrospinal fluid (CSF) of MS patients is of special interest.

In the present study we have compared a relative content of total protein and IgGs in sera and in CSFs of MS patients. Using different approaches, for the first time, we produce very strong direct evidence that amylase activity is intrinsic to IgGs from CSF of MS patients and we compare other parameters characterizing CSFs and sera of MS patients.

## Results

Fifteen patients (11 women and 4 men) satisfying the criteria for clinically or laboratory-supported definite MS according to [42, 43] were retrospectively selected for the study. Of these, 13 were relapsing–remitting (RR), and 2 were primary progressive (PP) in agreement with the criteria of Lublin and Reingold [44]. Clinical course (RR and PP), clinical activity (relapse at time of sampling), and MRI activity (the presence of gadolinium enhancing lesions at MRI examination) were analyzed as previously described [45]. The characteristics of the MS patients are summarized in Table 1. It should be mentioned, that after isoelectrofocusing all 15 preparations of Abs demonstrated from three to seven protein bands corresponding to oligoclonal IgGs.

**Table 1:** Several different characteristics of MS patients.

Number of patient	Sex	Age, years	Clinical course*	Clinical activity**	MRI activity^ξ^
1	male	59	PP	yes	yes
2	female	28	RR	no	no
3	female	36	RR	yes	yes
4	male	26	RR	yes	no
5	male	49	RR	no	no
6	female	20	RR	yes	no
7	female	46	PP	yes	no
8	female	51	RR	yes	yes
9	female	31	RR	yes	no
10	female	26	RR	no	no
11	female	43	RR	yes	yes
12	male	45	RR	yes	no
13	female	30	RR	no	yes
14	female	60	RR	yes	no
15	female	34	RR	yes	yes

*Relapsing–remitting (RR) and primary progressive (PP) MS.

**Clinical activity = presence of relapse at the time of sampling.

^ξ^MRI activity = presence or absence gadolinium enhancing lesions at MRI examination.

The relative concentrations of total protein of CSFs (range 0.26–0.66 mg/ml) and sera (47–74 mg/ml) of fifteen MS patients varied in different ranges. The average concentration of total protein in the serum (62.0±6.7 mg/ml) was ~129-fold higher compared with CSF (0.48±0.09 mg/ml; Table 2) and these values did not demonstrate good correlation (correlation coefficient (CC) = −0.12). The relative concentration of total IgGs in serum preparations (range 7.9–16.6 mg/ml; average value 11.7±1.8 mg/ml) was 195-fold higher than that for the CSF (range 0.02–0.14 mg/ml; average value 0.06±0.03 mg/ml) and there was not good correlation between these values, CC = +0.07. Interestingly, the concentration of total protein in CSF was 8-fold higher than total IgGs, while this difference in the case of serum (~5.3-fold) was by a factor of approximately 1.5 lower.

**Table 2:** The relative concentration of total proteins and total IgGs purified from sera and CSF of different MS patients*.

Number of patient	Relative concentration of total proteins, mg/ml		Relative concentration of total IgGs x 10^2^, mg/ml	
	CSF	Serum	CSF	Serum
1	0.55**	59	11.4	1190
2	0.33	63	1.9	925
3	0.26	72	3.2	1280
4	0.51	63	6.0	1160
5	0.64	48	6.4	1060
6	0.58	64	4.6	1140
7	0.56	64	11.3	1080
8	0.66	74	13.6	1450
9	0.37	73	7.5	1660
10	0.39	60	3.4	1010
11	0.42	58	2.4	1490
12	0.53	77	5.0	995
13	0.39	57	2.5	1000
14	0.47	58	9.0	1260
15	0.48	47	2.2	787
Average value	0.48±0.09	62.0±6.7	6.0±3.1	1166±178

*For each value, a mean of three measurements is reported; the error of the determination of values did not exceed 7–10%.

**Average values are reported as mean ± S.E.

**Fig 1:**
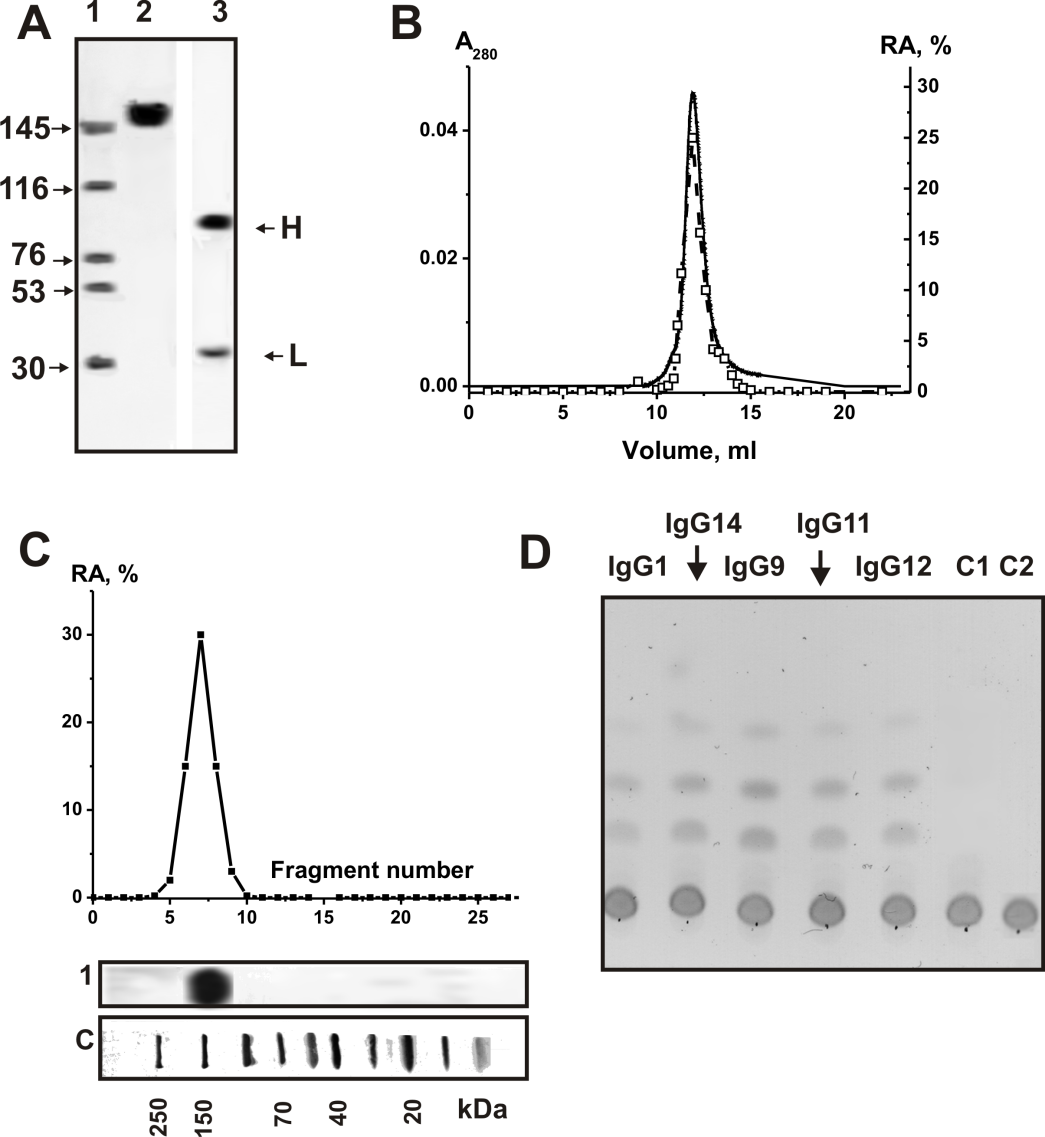
SDS-PAGE analysis of homogeneity of csf-IgG_mix_ (7 μg; lanes 2 and 3) corresponding to 15 CSFs of MS patients in 3–16% gradient gel before (lane 2) and after treatment with DTT (lane 4) followed by silver staining (A). Lanes 2 and 3 correspond to Western-blotting; Abs against human amylase were used in the case of IgG (lane 2) and human amylase (lane 3). The arrows (lane SP) indicate the positions of molecular mass markers. FPLC gel filtration of csf-IgG_mix_ on a Superdex 200 column in an acidic buffer (pH 2.6) destroying immunocomplexes after Abs incubation in the same buffer (B): (—), absorbance at 280 nm (A_280_); (□), relative activity (RA) of IgGs in the hydrolysis of MHO. A complete hydrolysis of MHO was taken for 100%. In-gel assay of MHO-hydrolyzing activity of csf-IgG_mix_ (■; 15 μg) of MS patients. The relative MHO -hydrolyzing activity (RA, %) was revealed using the extracts of 2-3-mm fragments of one longitudinal slice of the gel. The RA of IgGs corresponding to complete hydrolysis of MHO was taken for 100%. The second control longitudinal slice of the same gel was stained with Coomassie Blue (lane 1). Lane C shows positions of protein markers. TLC analysis of the hydrolysis of MHO by IgGs from CSFs of different MS patients (D). MHO (1.67 mM) was incubated for 6 h at 37°C without Abs (lanes C2) and in the presence of 0.07 mg/ml IgG_mix_ from the plasma of healthy donors (lane C1) as well as individual IgGs (0.025 mg/ml, 2 h) from CSFs of different MS patients (lanes 1–5). The average error in the initial rate determination from three experiments did not exceed 7–10%. For details, see Materials and methods.

It was previously shown, that individual IgGs and IgMs isolated from patients with clinically definite diagnoses of MS and SLE had approximately three orders of magnitude higher specific amylolytic activity than that for healthy donors [34–38]. In this work, similarly to [35–38], electrophoretically and immunologically homogeneous IgGs were purified from CSFs and sera from MS patients as well as from 10 healthy donors by sequential chromatography on protein A-Sepharose under conditions that remove non-specifically bound proteins, followed by gel filtration in an acidic buffer destroying immune complexes. The homogeneity of the 150-kDa csf-IgG_mix_ and ser-IgG_mix_ (equal amounts of electrophoretically homogeneous IgGs from 15 preparations of CSFs and sera, denote these preparations as respectively csf-IgG_mix_ and ser-IgG_mix_) as well health-IgG_mix_ (mixture of IgGs from 10 healthy volunteers) was confirmed by SDS-PAGE with silver staining, which showed a single band under non-reducing conditions and two bands corresponding to the heavy and light chains after reduction (for example, Fig 1A). Ser-IgG_mix_ and health-IgG_mix_ were also electrophoretically homogeneous. These IgG_mix_ preparations were described earlier [32, 33].

First, it was shown that all individual and mixed IgG preparations from CSFs (csf-IgG_mix_) and sera (ser-IgG_mix_) efficiently hydrolyze maltoheptaose (MHO) (see below). Then, electrophoretically homogeneous MS csf-IgG_mix_ preparation was used to prove that amylase activity of IgGs from the CSF of MS patients is an intrinsic property of these Abs [34–37]. Similarly to previous publications [14–17, 46], we here applied several rigid criteria for analysis of MS csf-IgG_mix_. The most important of these criteria are given below: a) electrophoretic homogeneity of MS csf-IgG_mix_ and ser-IgG_mix_ (Fig 1A). It was shown that Abs against human amylase give positive answer only in the case of amylase (lane 3), but not with IgGs (lane 2); b) FPLC gel-filtration of csf-IgG_mix_ under conditions of “acidic shock” (pH 2.6) did not lead to a disappearance of the activity, and the peak of amylolytic activity tracked exactly with 150 kDa IgGs (the amylolytic activity is absent in zones corresponding to molecular masses of known canonical amylases, 56–60 kDa [47] (Fig 1B); c) complete adsorption of the amylase activity by anti-IgG Sepharose leading to a disappearance of the catalytic activity from the solution and its elution from the adsorbent with buffer of acidic pH. In order to confirm previously obtained data [35–38], similar results were obtained for MS ser-IgG_mix_.

To exclude possible artifacts due to hypothetical traces of contaminating canonical enzymes, the MS csf-IgG_mix_ preparation was separated by SDS-PAGE and its amylase activity was detected after the extraction of proteins from the separated gel slices (Fig 1C). Since SDS dissociates any protein complexes, and the electrophoretic mobility of usually low molecular mass amylases (56–60 kDa) cannot coincide with that of intact IgGs (or Ab complexes with amylases), the detection of amylolytic activity in the gel region corresponding only to intact IgGs from CSFs (together with absent of a positive answer of IgGs with Abs against human amylase) provides direct evidence that CSF IgGs do not contain admixtures of canonical amylases, but they possess amylolytic activity.

Fig 1 illustrates typical examples of maltoheptaose cleavage by individual IgGs from CSF of several MS patients. In order to estimate amylase relative activity (RA) quantitatively, we found the concentration for each preparation IgG (total serum or CSF protein) and the time of incubation sufficient to convert maltoheptaose into products of the hydrolysis (10–40%; for example, lanes IgG1 and IgG12 of Fig 1D).

The activities of IgG preparations were determined as a decrease in the percentage of initial maltoheptaose corrected for the distribution of different oligosaccharides between the bands in the control. Since all measurements (initial rates) were taken within the linear regions of the time courses and protein concentration, the measured RAs were normalized to standard conditions (mmole maltoheptaose /1 mg of IgGs (or protein)/1 h; standard activity units (AU)) (Table 3).

**Table 3:** Relative amylase activity of total proteins and IgGs from sera and CSFs of patients with MS*.

Number of patient	1. Activity of plasma protein; (mM/1 h/1 mg)	2. Activity of IgGs from the plasma; (mM/1 h/mg) ×10^3^	3. Activity of CSF protein; (mM/1 h/1 mg)×10^3^	4. Activity of IgGs from CSF; (mM/1 h/mg) ×10^3^
1	0.30	0.73	1.1	6.0
2	0.32	0.23	0.39	2.5
3	0.36	0.29	0.35	3.1
4	0.32	0.57	0.89	5.1
5	0.24	0.08	0.33	1.8
6	0.32	0.10	0.62	7.4
7	0.32	0.09	0.67	8.4
8	0.37	0.2.	0.89	2.4
9	0.37	0.24	0.46	17.1
10	0.30	0.18	0.62	7.1
11	0.29	0.24	0.40	17.0
12	0.39	0.23	0.64	13.8
13	0.29	0.38	0.52	19.0
14	0.29	0.46	0.55	11.8
15	0.24	0.37	0.6	11.9
Average values	0.31±0.03	0.30 ±0.14	0.6 ±0.16	9.0 ± 4.9
Ratio of all values	**1/2**: 1033; **1/3**: 516; **1/4**: 34.4; **2/3**: 0.5; **2/4**: 0.033; **3/4**: 0.066			
Coefficient correlation	**1/**2: −0.03;**1/3**: 0.18; **1/4**: 0.07; **2/3**: 0.7; **2/4**: 0.03; **3/4**: −0.19			

*For each value, a mean of three measurements is reported; the error of the determination of each value does not exceed 7–10%.

In principle, sera and CSF may contain not only amylolytic IgGs, but also canonical amylases. The relative specific amylase activity of the total protein of sera preparations at a fixed concentration of MHO (1.67 mM) was varied in the range 0.24–0.37 mmole MHO/1 mg of protein/1 h (average value 0.31 ± 0.03 AU). It was surprising that the specific activity of the total protein of CSF reparations was ~516-fold lower (range (0.33–1.2)×10^−3^ AU, average value (0.6 ±0.16) ×10^−3^ AU) than the serum ones and the correlation coefficient (CC) between RA values was positive, but low, +0.18 (Table 3).

Among 15 individual MS patients, the RAs of IgGs from sera were very different; the specific RAs varied in a range (0.08–0.73) ×10^−3^ AU. The average RA value was (0.30±0.14)×10^−3^ AU and it was 1033-fold lower than that for the total protein of the serum; CC between these RA values was very low and negative, −0.03 (Table 3).

The specific RAs of the CSF IgGs varied in a range (1.8–19.0)×10^−3^ AU; the average value of RAs ((9.0 ± 4.9)×10^−3^ AU) was by a factor of 15 higher than that for the total protein of CSF (Table 3). It was surprising, but the average specific RA of IgGs from CSFs was about 30-fold higher than that for IgGs from serum preparations. The CCs between RAs of the CSF IgGs and relative activities corresponding to total protein of CSF (−0.19) and IgGs from serum (0.03) were low. The detectable hydrolysis of MHO by IgGs from healthy donors was observed for six of ten Ab preparations at a 10–40–fold higher concentration and during a significantly longer time of incubation (12–36 h). Average RA for IgGs from healthy volunteers was approximately 1000 lower than that for MS patients.

## Discussion

No analyses were made before the amylase activity of Abs from CSF. Data reported in this paper provide strong evidence that amylase activity is an intrinsic property of IgGs present in CSF of MS patients: it is not due to copurifying enzymes. At entry, none of the patients or donors had symptoms of infections. It was shown that CSFs and sera as well as IgGs after purification do not contain any bacterial or canonical enzymes contaminations (Fig 1). Csf-IgG_mix_ and ser-IgG_mix_ showed amylolytic activity after FPLC gel-filtration under conditions of “acidic shock” and SDS-PAGE in zones corresponding only to intact IgGs; there was no activity corresponding to zones of canonical amylases having relatively lower molecular masses than IgGs. In addition, IgGs from only six of ten healthy donors demonstrated detectable amylase activity; the average RA was ~1000-fold lower.

Overall, abzymes of MS patients may be significantly more active in the hydrolysis of maltoheptaose than what we found (Table 3). As previously shown by us, the fraction of abzymes with different catalytic activities, including the nuclease, protease and amylase ones, in serum of autoimmune patients usually does not exceed 1–7% of total immunoglobulins [14–17]. Since the specific activity was calculated using the total concentration of IgGs, the specific amylase activities of the individual monoclonal subfractions in a polyclonal IgG pool may be significantly higher than those of the non-fractionated IgGs. In addition, the repertoire of polyclonal Abs against different antigens in the case of sera from MS patients may be significantly wider than that of CSFs. It may be one of the possible reasons of a lower specific activity of serum IgGs.

At the same time, an ever-growing number of observations suggest that autoimmune diseases originate from defects in hematopoietic stem cells [48]. It has recently been shown that the specific reorganization of the immune system during spontaneous development of a profound SLE-like pathology in MRL-lpr/lpr mice [49–51] and MS-like pathology in experimental autoimmune encephalomyelitis mice [31] is associated with changes in the differentiation profile, the level of proliferation of bone marrow hematopoietic stem cells and the production of DNase, protease, ATPase, and amylase abzymes. Immunization of healthy mice with DNA also leads to a production of Abs with DNase activity; however, it is only associated with increased lymphocyte proliferation and suppression of apoptosis of lymphocytes in different organs (especially in the spleen), but not with a change in the differentiation of bone marrow cells [49–51]. Thus, it is reasonable to suggest that B-cells of CSF of MS patients can produce not only Abs interacting with oligosaccharides, but abzymes with amylase activity. Abzymes produced by lymphocytes in different organs of MS patients (and circulating in the blood system) may have a lower amylase activity in comparison with Abs of CSF, or there may be different ratio of abzymes and Abs without catalytic activity in the CSFs and sera of MS patients.

It should be mentioned, that IgGs and sIgAs from breast milk [52] and IgGs from sera of MRL-lpr/lpr mice [50, 51] demonstrated high ATPase activity. It was surprising, but individual IgGs from sera and CSF of MS patients did not possess detectable ATPase activity (even after reaction mixtures containing 0.5 mg/ml Abs incubation during 48 h).

We have not revealed high correlation coefficients between different RAs characterizing IgGs of CSF and serum as well as RAs of these IgGs and total proteins corresponding CSF and serum (Table 3). All CCs varied from −0.03 to +0.18 except unpredicted correlation of RAs for IgGs from serum and total protein corresponding to CSF (CC = 0.7). Similar situation was observed earlier for CCs between RAs of these IgGs and total proteins corresponding CSF and serum in the hydrolysis of DNA (CCs = −0.05–+0.03) [32].

Using the data of Table 3, we calculated apparent *k*_*cat*_ = V / [IgG], characterizing IgGs from serum and CSF at a fixed concentration of MHO (Table 4). In addition, we calculated a possible correlation between these values and *k*_*cat*_ characterizing 15 IgG preparations in the hydrolysis of DNA and MBP (Table 4). All CCs were relatively low (−0.009–+0.2) except some of them: IgG amylase activity of CSF correlates with MBP-hydrolyzing activity of serum (CC = +0.41) and CSF (CC = +0.45); DNase activity of serum IgGs correlates with MBP-hydrolyzing activity of serum (CC = +0.44) and CSF (CC = +0.61), as well as MBP-hydrolyzing activity of serum and CSF (CC = +0.59). However, all the correlation coefficients were very low or relatively low. Thus, an additional question is why there is no good correlation between various indexes, characterizing different MS patients.

**Table 4:** Relative *k*_cat_ values and correlation coefficients between these values of 15 IgGs from plasma and CSFs in the hydrolysis of MHO, DNA, and MBP*.

Number of patient	Amylase activity of plasma IgGs; k_cat_, min^−1^	Amylase activity of CSF IgGs; k_cat_, min^−1^	DNase activity of plasma IgGs x 10^3^; k_cat_, min^−1^	DNase activity of CSF IgGs x 10^2^; k_cat_, min^−1^	MBP-hydrolyzing activity of plasma IgGs x 10^3^; k_cat_, min^−1^	MBP-hydrolyzing activity of CSF IgGs; k_cat_, min^−1^
Parameter number	1	2	3	4	5	6
1	1.84	15.4	2.7	8.0	1.7	0.33
2	0.58	6.3	0.79	10.9	0.51	0.044
3	0.73	7.8	0.54	11.8	2.0	0.18
4	1.44	12.9	1.7	8.2	5.2	0.17
5	0.20	4.5	1.6	8.5	0.44	0.008
6	0.25	18.7	1.7	8.2	14.0	0.1
7	0.23	21.2	2.0	8.2	5.9	0.26
8	0.51	6.1	2.7	2.0	5.6	0.53
9	0.61	43.2	2.6	1.8	6.8	0.6
10	0.45	17.9	1.2	3.2	6.1	0.3
11	0.61	42.9	3.2	18.0	14.0	0.51
12	0.58	34.8	1.8	14.0	10.0	0.72
13	1.0	48	0.59	2.6	0.44	0.06
14	1.16	29.8	1.8	9.5	16.0	0.6
15	0.93	30.0	1.6	8.3	7.0	0.81
Average values	0.74±0.36	22.6±12.4	1.77±0.59	8.2±3.1	0.0064±0.0042	0.35±0.22
Correlation coefficient	**1/2:** 0.22; **1/3**: 0.03; **1/4:** −0.009; **1/5:** −0.13; **1/6**: 0.1; **2/3:** 0.22; **2/4:** 0.03; **2/5:** 0.41; **2/6:** 0.45; **3/4**: 0.11; **3/5:** 0.44; **3/6:** 0.61; **4/5**: 0.34; **4/6:** 0.11; **5/6:** 0.59					

*For each value, a mean of three measurements is reported; the error of the determination of each value does not exceed 7–10%.

An analysis of correlation between titers of Abs to DNA as well as to MBP and 13 different standard clinical parameters including Poser criteria (indexes for evaluation of damage to functional systems: pyramidal functions; cerebellar functions; functions of brain stem; sensitive functions; functions of intestines and urinary bladder; visual functions; cerebral (psychical) functions and sum of these characteristics) in the case of 49 patients with MS, was carried out [14]. For the whole group of MS patients, the absolute values of positive CCs between titers of anti-DNA or anti-MBP Abs and clinical Poser indexes were very low (between 0.01 and 0.19), absent (~0), or even negative (−0.02 to −0.07) and statistically non-significant. Several CCs became higher and reached values up to 0.1 to 0.55 and –0.04 to –0.47 after the division of cohort into subgroups of patients with primary progressing, secondary progressing and remitting course of the disease [15].

The groups of primary progressing remitting course and secondary progressing course of MS patients were not “homogenous” with respect to the patients’ characteristics, and their further subdivision using cluster and factorial analysis revealed high statistically significant correlation coefficients [15]. For example, for one sub-subgroup of the remitting course subgroup, a direct dependence between titers of anti-MBP and symptoms of lesions of the pyramidal tract could be observed (CC = 0.92). In some cases, correlations of the opposite sign were observed for the same pairs of analyzed parameters for the three subgroups with different MS courses and in their sub-subgroups obtained by cluster analysis from the subgroups.

The absence of a definite dependence between titers of anti-DNA and anti-MBP Abs and these parameters with standard clinical indices may be caused by several reasons. MS is an extremely multifactorial disease, in which similar pathomorphological and clinical indices manifested as MS may result from very different underlying processes and conditions [53, 54]. For example, in each MS patient, the “relative stability” of different organs and their functions to the destructive effect of transient immune system errors can be significantly different depending on the genetic background and environmental stress factors, including geographic ones [53, 54]. Some proteins of influenza, herpes, polyoma, Epstein–Barr and other viruses and of some bacteria have been reported to mimic human myelin proteins, and these infections can therefore lead to immunization with their proteins and stimulate the subsequent formation of Abs to myelin and finally to the development of autoimmune reactions. In individual MS patients, the development of autoimmune reactions can be stimulated by different viral or bacterial infections as well as various toxic chemicals. Furthermore, it should also be taken into account that MS is a pathology of at least two-phases [55]. The cascade of reactions corresponding to the first inflammatory phase is very complicated and involves many proteins, enzymes, cytokines, and chemokines inducing macrophages and other cells producing NO● radicals and osteopathin [55]. The complex and coordinated action of T- and B-cells, complement system, inflammation mediators-, and auto-Abs result in the formation of demyelinization nodi and the interruption of axon conductivity. The neurodegenerative phase of MS that ensues thereafter is directly connected to the neural tissue destruction in these patients [53–55]. Therefore, any analysis of biochemical, immunological and clinical indices must take into account the current stage of the disease. Obviously, quite different characteristics of pathologic processes can be obtained in individual patients as the disease progresses against the background of the continually changing immunoregulation, including the exhaustion of different compensatory and adaptive mechanisms and systemic metabolic changes. This makes the clinical course of MS hardly predictable in individual patients [53–55]. Therefore, it is not surprising that we could not find a statistically significant correlation of titers of Abs to MBP and DNA as well as RAs of abzymes with all parameters measured, since each patient can be characterized by an individual combination of genetic, environmental, chronic, inflammatory, autoimmune, demyelinating, neurodegenerative and/or other factors.

In general, all data obtained demonstrate that the amylase activity is an intrinsic property of IgGs deriving from CSF and sera of MS patients. It is extremely unpredictable result showing that average amylolytic activity of IgGs from CSFs is 30-fold higher, than that for IgGs from sera. This result correlates with previously published data: average DNase and MBP-hydrolyzing activities of IgGs from CSFs are respectively 49- and ~54-fold higher than those for abzymes from sera [32, 33]. It seems reasonable to suggest, that CSF cells can produce abzymes with higher enzymatic activity than those of blood sera.

We have previously shown that the appearance of abzymes specifically hydrolyzing DNA, MBP, and significant increase in amylase activity is among the earliest and clearest signs of autoimmune reactions in a number of autoimmune diseases when titers of Abs to various auto-antigens have not yet increased significantly and correspond to their ranges for healthy donors [14–17, 49–51]. Abs of healthy humans are inactive in the hydrolysis of DNA and MBP, while they possess low amylolytic activity Therefore, detection of abzymes with DNase-, MBP-hydrolyzing activities in combination with a significant increase in the oligosaccharide-hydrolyzing activity of Abs form sera and CSF of people can be considered as an additional criterion (immunological parameter) for early diagnostics of MS. Taking the data in account, we propose that MBP-hydrolyzing, DNase, and polysaccharide-hydrolyzing abzymes may, in addition to other factors, cooperatively promote important neuropathologic mechanisms in MS and SLE pathogenesis.

## Methods

### Patients, donors and chemicals

Most chemicals, maltoheptaose, Protein G-Sepharose, and the Superdex 200 HR 10/30 column were from Sigma or GE Healthcare.

Fifteen consecutive MS patients (11 women and 4 men; mean age = 39 ± 12.5 years), satisfying the criteria for definite MS according to the classification of McDonald [42] and admitted to the Multiple Sclerosis Center of the University of Ferrara during the period that goes from January 2012 to October 2012, were retrospectively selected for the study. Disease severity was scored in all MS patients at the time of sample collection using Kurtzke’s Expanded Disability Status Scale (EDSS) [43] (mean at entry = 1.8 ± 1.4; range from 0 to 4.0). Clinical course (RR and PP), clinical activity (relapse at time of sampling), and MRI activity (the presence of gadolinium enhancing lesions at MRI examination) were analyzed as previously described [45]. In addition, we used IgGs from blood serum of healthy donors for control. At entry none of the patients or donors had fever or other symptoms or signs of acute infections. Moreover, at the time of sample collection none of the patients had received any potential disease-modifying therapies during the 6 months before the study. These 15 patients had previously been used by us for the analysis of DNase [32] and MBP-hydrolyzing activity [33] of IgGs.

### Sample preparation

Blood and CSF sampling protocols were confirmed by the local committee for medical ethics in research (Comitato Etico della Provincia di Ferrara) that approved our study in accordance with the Helsinki ethics committee guidelines including a written consent of patients confined to present of their blood and CSF for diagnostics of a possible disease and scientific purposes. The protocol was approved on May 31 2007 and it was focused on the creation of a biological bank of CSF and serum samples, and related clinical data of patients with MS and other neurological diseases including: a) a study of potential markers (especially proteins) for diagnostic and prognostic significance in diseases of the nervous system; b) specific antibodies directed against antigens, potential exogenous and / or endogenous; c) presence of pathogens (mostly viruses or bacteria) for association studies and pathogenesis; d) neurotransmitters and their metabolites; e) a study of different properties of different markers.

All participants provided their written consent before inclusion and the study design was approved by the Regional Committee for Medical Ethics in Research.

CSF and serum samples were collected under sterile conditions and stored in aliquots at –80°C until assay. “Cell-free” CSF samples were obtained after centrifugation, at room temperature, of specimens obtained by atraumatic lumbar puncture performed for diagnosis purposes in the absence of contraindications. Serum samples derived from the centrifugation of blood specimens withdrawn by puncture from an anterocubital vein at the same time of a CSF extraction. Paired CSF and serum samples from MS patients were stored and measured under exactly the same conditions. Informed consent was given by all patients before inclusion and the study design was approved by the Regional Committee for Medical Ethics in Research. CSF and serum IgG levels were measured by immunochemical nephelometry with the Beckman Immage 800 Immunochemistry System (Beckman Instruments, Inc. Fullerton, CA. USA) according to the procedure of Salden et al. [56].

### Analysis of protein concentrations

In all cases, protein concentration in the intact CSF, sera of MS patients and final solutions of Abs was measured using Bradford assay with a bovine serum albumin standard.

### Analysis of the concentration of total IgGs

Relative concentrations of total IgGs in the intact CSF and in sera of MS patients were analyzed using a special quantitative isoelectrofocusing and immunoblotting test system according to the standard manufacturer’s protocol and equipment (IgG IEF, Helena Laboratories, Gateshead, Tyne and Wear, UK). Concentration of IgGs (mg/ml) was estimated using calibration curves obtained according to the standard manufacturer’s protocol.

### IgG purification

Electrophoretically and immunologically homogeneous IgGs were obtained by sequential affinity chromatography of the CSF and serum proteins on protein A-Sepharose and FPLC gel filtration similarly to [32, 33]. In order to protect the Ab preparations from bacterial contamination they were sterilized by filtration through a Millex filter (pore size 0.2 μm). In each case the protein corresponding to the central part of IgG peaks was concentrated in sterile condition and used in further purification or analysis. Incubation of standard bacterial medium with initial non-fractionated preparations of the sera, CSFs, and stored Ab preparations did not lead to a formation of colonies.

IgGs from CSF were incubated in 50 mM glycine-HCl (Ph 2.6) containing 0.2 M NaCl for 20 min. at 25°C. Separation of the IgGs under “acid shock” conditions was carried out by FPLC gel filtration on a Superdex 200 HR 10/30 column equilibrated with 50 mM glycine-HCl (pH 2.6) containing 0.1 M NaCl as previously described [32, 33]. After 1–2 weeks of storage at 4°C, in order to refold Abs after the acid shock, these Abs were used in the activity assays described below.

In some cases, electrophoretically homogeneous IgGs were chromatographed on Sepharose bearing immobilized polyclonal mouse IgGs against human IgGs. The protein was applied to the column (1 ml) equilibrated with 20 mM Tris-HCl (pH 7.5) containing 0.1M NaCl and the column was washed with the same buffer containing 0.3 M NaCl. Abs were eluted in 0.1 M glycine-HCl (pH 2.6), neutralized, dialyzed and sterilized as described above [32, 33]. All preparations from the serum and CSF of MS patients and from healthy donors were used previously for analyses of DNase and MBP-hydrolyzing activity [32, 33].

### Amylase activity assay

Amylase activity was analysed as in [49, 50]. The reaction mixture (20 μl) containing 50 mM Tris-HCl, pH 7.5, 1 mM NaN_3_, 1.67 mM MHO and 0.001–0.2 mg/ml of IgGs was incubated for 1–6 h at 37°C. In the case of IgGs from sera of healthy donors we used a higher concentration of IgGs (0.25–0.5 mg/ml) and a longer time of incubation (12–36 h). Products of hydrolysis were identified by TLC on Kieselgel plates (Merck) using 1-butanol-acetic acid-H_2_O (12:4:4). The activities of IgGs were determined from the scanning data as a relative percentage of oligosaccharide in the spots of MHO and its hydrolyzed forms. All measurements were taken within the linear regions of the time courses and Ab concentration curves. If the Ab activity was low (< 5–10% of hydrolysis) the incubation time was increased to 5–36 h. If MHO hydrolysis exceeded 40%, the concentration of Abs was decreased 2-100-fold depending on the sample analyzed. Finally the catalytic RAs were normalized to 1 mg/ml IgGs and 1 h of incubation.

### SDS-PAGE assay of amylolytic activity

SDS-PAGE analysis of Abs for homogeneity and for the polypeptide spectrum of sera and CSF was performed in a 5–16% gradient gel containing 0.1% SDS (Laemmli system) as described in [32, 33]. The polypeptides were visualized by silver and Coomassie Blue staining [32, 33]. In addition, Western-blotting analysis of IgGs interaction with Abs against humans amylase was performed using specific Western-blotting test system from Abcam (United Kingdom), human amylase was used as positive control (Fig 1).

Analysis of amylolytic activity of MS IgGs from CSF and sera after SDS-PAGE was performed similarly to the analysis of the amylolytic and proteolytic activities of different abzymes [18–21, 37]. IgGs (10–40 μg) were pre-incubated at 30°C for 30 min under nonreducing (50 mM Tris-HCl, pH 7.5, 1% SDS, and 10% glycerol) condition. After standard SDS-PAGE electrophoresis of Abs to restore the amylase activity of IgGs, SDS was removed by incubation of the gel for 1 h at 30°C with 4 M urea and washed 10 times (7–10 min) with H_2_O. Then 2-3-mm cross sections of longitudinal slices of the gel were cut up and incubated with 50 μl 50 mM Tris-HCl, pH 7.5, containing 50 mM NaCl for 6 days at 4°C to allow protein refolding and eluting from the gel. The solutions were removed from the gels by centrifugation and used for assay of MHO hydrolysis as above described. Parallel control longitudinal lanes were used for detecting the position of IgG on the gel by Coomassie R250 staining.

### ATP-hydrolyzing activity assay

ATP-hydrolyzing activity was analyzed as in [50, 51]. Reaction mixtures (10–20 μl) 5 mM MgCl_2_, 1 mM EDTA, 50 mM Tris-HCl, pH 7.5, 2 mM ATP and 0.2–0.5 mg/ml IgG. The reaction mixtures were incubated for 12–36 h at 37°C. The products of ATP hydrolysis were analyzed by thin-layer chromatography in 0.25 M KH_2_PO_4_ (pH 7.0) on PEI-cellulose plates (Merck) using the system dioxane—10% NH_4_OH- Н_2_O (6:1:4). After chromatography, the plates were dried and the positions of various products were identified. The relative amount of products was calculated using scanning data.

### Statistical analysis

The results are reported as mean ± S.E. of at least three independent experiments for each sample analyzed. Errors in the values were within 7–10%. The correlation coefficients (CC) between sets of different samples were analyzed using Statistica 10 (StatSoft Inc.). The differences between samples were analyzed by the Student’s *t*-test, *p* < 0.05 was considered statistically significant.
